# The effect of personal relative deprivation on food choice: An experimental approach

**DOI:** 10.1371/journal.pone.0261317

**Published:** 2022-01-13

**Authors:** Sofie van Rongen, Michel Handgraaf, Maaike Benoist, Emely de Vet

**Affiliations:** 1 Consumption and Healthy Lifestyles Group, Wageningen University & Research, Wageningen, Gelderland, The Netherlands; 2 Urban Economics Group, Wageningen University & Research, Wageningen, Gelderland, The Netherlands; 3 Human Nutrition and Health Group, Wageningen University & Research, Wageningen, Gelderland, The Netherlands; University of Suceava: Universitatea Stefan cel Mare din Suceava, ROMANIA

## Abstract

Growing evidence suggests that relative disadvantage is more relevant than absolute socioeconomic factors in explaining disparities in healthfulness of diet. In a series of pre-registered experiments, we tested whether personal relative deprivation (PRD), i.e. the sense that one is unfairly deprived of a deserved outcome relative to others, results in choosing more palatable, rewarding foods. Study 1 (*N* = 102) demonstrated the feasibility and effectiveness of a game for inducing real-time experiences of PRD. Study 2 (*N* = 287) showed no main effect of PRD condition on hypothetical food choices, but an interaction between chronic PRD and condition revealed that those in the PRD condition chose more rewarding foods when feeling chronically deprived. In Study 3 (*N* = 260) the hypothesized main effect was found on real, non-hypothetical food choices: those in the PRD condition chose more rewarding foods, controlling for sensitivity to palatable food. Our results provide preliminary indications that the experience of being relatively deprived, rather than the objective amount or resources, may result in a higher preference for high-caloric and palatable foods. It may be suggested that efforts to reduce societal disparities in healthfulness of diet may need to focus on perceptions of injustice beyond objective inequalities.

## Introduction

The association between socioeconomic status (SES) and diet quality is globally well established [1,2]. People living on a low income have unhealthier diets [3] and higher rates of diet-related diseases such as obesity [4,5] than people of higher SES. Dominant explanations for socioeconomic disparities in diet and obesity have focused on physical and economic food access. Unhealthy food outlets are more prevalent in low SES neighbourhoods [6], and it has been claimed that unhealthy foods are cheaper than healthy alternatives [7]. Importantly, having a low income or a low educational status in an absolute sense does not fully explain socioeconomic inequalities in diet. Inequality also comprises a relative aspect, i.e. earning less than others or being less educated than others. Relative disadvantage may even be more relevant than absolute factors like income. This is illustrated with epidemiological evidence showing a positive correlation between societal income inequality and obesity prevalence [8,9], even after controlling for absolute socioeconomic measures [10,11]. Additionally, growing evidence shows that subjective, relative wealth is indeed more predictive of health than objective, absolute socioeconomic indicators [12]. The relative deprivation hypothesis proposes that making upward comparisons has negative psychological consequences, leading to health compromising behaviour. Focusing on dietary behaviour specifically, a correlational study showed that the Yitzhaki index, a demographic measure of relative deprivation (income), was associated with self-reported behaviours such as less healthful food choices [13]. However, evidence for a causal relation between relative deprivation and diet quality at the proximate, individual level is lacking. In a series of experimental studies, we aimed to address this gap by experimentally inducing relative deprivation and investigating how it affects food choice behaviour.

A common conceptualization of subjective, individual-level relative deprivation is personal relative deprivation (PRD), which relates to feelings of frustration and resentment in response to the idea of being deprived of a deserved and desired outcome, stemming from upward comparisons with similar others [14–16]. Human concern for justice is a key prerequisite for the experience of relative deprivation [17]; a threat to one’s personal deservingness produces perceptions of injustice and unfairness [14,15,18]. PRD has been associated with various adverse outcomes, including depression [19], physical and mental health issues [20], but also gambling and other risk behaviours [21,22]. As a psychological mechanism, feelings of PRD have been theorized to result in a greater desire for immediate small rewards The reasoning for this, drawing on theories of justice motivation [23] and delay discounting [24], is that people who experience feelings of not being treated in the same way as others prefer immediate small rewards because of the need to feel that their deservingness concerns are being addressed [21]. If people lose their trust in a just world, then they might be more attracted to immediate gratification at the expense of longer-term, larger gains [23,25].

We posit that, in the current food context of easy food access and abundant choice, people experiencing PRD may similarly have a preference for foods that are immediately rewarding rather than beneficial for health, as a way to combat these negative experiences and restore the sense of personal deservingness. Although food may not be as rewarding as monetary rewards in response to deprivation of resources, recent research on resource scarcity-induced eating indicates that the human motivational system for food and money overlap [26–28]. Furthermore, the experience of PRD may be a precursor to a higher general sensitivity to reward, and it has previously been found that individual sensitivity to reward predicts a preference for high-calorie food [29,30]. Moreover, the motivational component of food reward is essentially driven by the brain’s appetitive system, largely the dopamine pathway, which facilitates ‘wanting’–the motivation to pursue a stimulus [31]. This pathway also mediates the motivation for risk behaviours like gambling [32,33]. Hence, PRD may evoke a pleasure-oriented preference for selecting high-fat or high-sugar, palatable foods, as these are, based on previous encounters, (implicitly) associated with immediate reward [34].

The present research links to a growing number of experimental studies that have demonstrated an effect of a subjective relative socioeconomic manipulation on high-caloric food preference and intake (see for reviews [35,36]. For example, Briers and Laporte [37] showed that a manipulation of relative income resulted in a higher selection of high versus low caloric dishes. Cheon and Hong [38] demonstrated that low subjective socioeconomic status (SSS) resulted in a greater preference for high-caloric foods (over fruits and vegetables). SSS was induced with a popular, much-used manipulation, i.e. an adapted version of the MacArthur Ladder of SSS [39–42]. Participants were asked to compare themselves with those who were at either the very top (low SSS condition) or the bottom (high SSS condition) of the ladder by indicating where they stood relative to these people, and to write about a hypothetical interaction with one these individuals.

Although relative income and SSS income involve a subjective evaluation of relative standing, they lack an important emotional component of relative deprivation. PRD is a more specific and emotionally laden concept of relative status that comprises both cognitive and affective responses to unfair outcomes, and has been shown to be a better predictor of health than relative SSS [43]. Whether PRD affects food preference as a proxy for diet quality remains to be tested, and with this research we aimed to answer this question.

One previous study provided first evidence for a causal effect of PRD on food selection, showing that induced feelings of PRD resulted in the selection of larger meal portions in a computerized portion selection task [44]. However, in that experiment, the PRD manipulation involved reading a hypothetical scenario unrelated to the food (receiving a smaller versus equal work bonus relative to colleagues) and the portion size selection was hypothetical in nature. The present study expanded on these first results in two main ways. First, it aimed to test the impact of real-time experience of PRD in resources on both hypothetical and actual food choices. Second, the resources earned were linked to food choices, as earnings served as resources to be spent on foods. This contributes to external validity because in actual life/natural environments eating is almost always a choice and foods are usually obtained with one’s resources (e.g. while grocery shopping), rather than by self-serving from free buffets, as commonly applied in experimental studies focusing on SES (although self-serving is a valuable measure in other regards, i.e. actual assessment of quantity consumed and portion control). In Study 1, we tested the feasibility of a self-developed card game for manipulating real-time experiences of PRD. In Study 2, we tested the effect of PRD on food preference in a hypothetical online food shopping setting. Following a pre-test, the available foods were categorized into rewarding and neutral foods. Study 3 was a conceptual replication of Study 2 in a lab-in-the-field setting, where a diverse community sample made real (non-hypothetical) food choices using the points earned. It was hypothesized that PRD would result in a higher selection of high-caloric and palatable (immediately rewarding) foods.

## Study 1: Testing the PRD manipulation task

The aim of Study 1 was to examine whether experiences of PRD can be effectively induced using a self-developed card game. In conformity with a pilot study in which participants were relatively disadvantaged in a Monopoly game [45], our PRD manipulation involved playing a computer card game in which participants actively experienced earning fewer (PRD condition) versus equal (control condition) resources relative to a fictitious player.

### Methods

#### Participants and procedure

On the basis of a power calculation (power of .90, medium effect size (Cohen’s *d*) of .5), 172 participants aged between 18 and 70 years with fluency in English were recruited online via Prolific, an online participant recruitment platform (www.prolific.com). Four participants were excluded and substituted with new participants because they completed the study in a substantially short amount of time (i.e. below 6 minutes). Another exclusion criterion was incorrect answers on both the attention check items (none excluded). The analytic sample consisted of 102 (59%) females, 15 different nationalities (51% British), an average age of 34.83 (*SD* = 11.39), and a range of educational backgrounds (24% no education/high school degree, 27% college/associate degree, 47% academic degree). All participants provided written informed consent at the start of the study. After the game, participants completed demographic measures and a “bogus” end task in which they allocated all of their earnings (30 points) amongst four food products (two healthy (radishes and carrots) and two unhealthy (waffles and pralines)), based on how much they would want the product. An analysis of this data showed that conditions did not differ in the amount of points allocated to healthy or unhealthy foods (see S3 Appendix for the specific results). Yet, as this task was under-developed at this stage and the main goal of this study was to test the PRD manipulation (for which a purpose for the earnings needed to be included), we consider these non-significant results unproblematic with respect to the ultimate outcome of interest (food choice). The Social Science Ethics Committee of Wageningen University approved the study.

#### PRD manipulation: Card game

The card game was designed to induce subjective experiences of relative deprivation, which specifically entail upward social comparisons leading to perceptions of being worse off and unfairly treated as well as feelings of resentment, dissatisfaction, and anger [14,21]. The card game was developed in Qualtrics, an online survey tool [46]. It was explained that the card game served as a way to earn points that were necessary for completing a subsequent task. The participants were led to believe that they were playing the game against a previous participant (a fictitious opponent) of the same gender and age, so as to induce an idea of a ‘similar other’. In each of 10 rounds, two different playing cards were presented (e.g. four of diamonds and nine of clubs). All participants were shown the exact same cards. Participants had to guess whether the number of a third card, supposedly drawn at random by the computer, would be between or not between the numbers on these cards.

**Fig 1:**
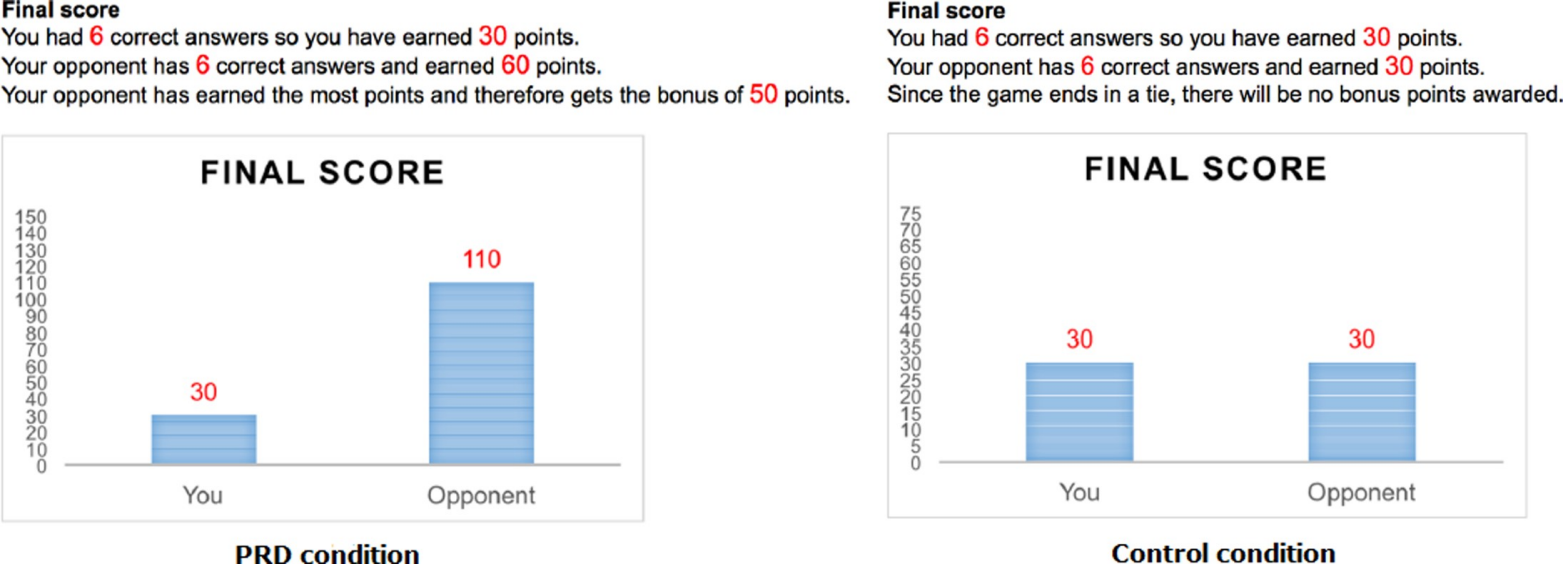
Screens of the final scores of the PRD condition and the control condition, as part of the PRD manipulation. The points earned did not objectively differ between conditions, conditions differed in the idea that the opponent earned much more (PRD condition) or just the same (control condition) for the same number of correct answers.

After a practice round, participants were randomly assigned to either the PRD or the control condition by Qualtrics. Before the actual game started, participants read an additional instruction page about the point earnings, which differed for the two conditions. Participants in the PRD condition learned that they would receive fewer points than their (fictitious) opponent for each correct answer (i.e. 5 versus 10 points) and fewer bonus points than their (fictitious) opponent if they, rather than their opponent, had most points at the end of the 10 rounds (i.e. 25 versus 50 bonus points). The participants in the control condition learned that would earn the same as their opponent for each correct answer (i.e. 5 points) and an additional 25 bonus points for having most points after 10 rounds. After each round, participants were shown a bar chart depicting the interim score of points earned by themselves and their opponent. For example, after round 1, participants in the control condition earned 5 points, the same as their opponent who also earned 5 points, whereas participants in the PRD condition earned 5 points while their opponent earned 10 points. Unbeknownst to the participant, the ‘game’ was completely pre-programmed, such that all participants (and the fictitious opponent) had a total of six correct answers (in round 1, 3, 4, 5, 8, and 10) and so *each* participant ended with total score of (6x5 =) 30 points. As the earning of bonus points was based on having the most points at the end of the game, the distribution of bonus points also differed between conditions (i.e. 50 points for the opponent of participants in the PRD condition and no bonus points for participants and their opponent in the control condition). Fig 1 presents the final score screens of the PRD and the control condition.

#### Assessment of experienced PRD

A 7-item scale was developed based on Callan, Shead, and Olson’s revised PRD scale [21] that asked about the perception of being deprived and unfairly treated (e.g. “I felt worse off when I compared myself with my opponent”), as well as feelings of resentment, dissatisfaction, and frustration (e.g. “I was frustrated when I saw how many points I earned compared to my opponent”). The answer scale ranged from 1 (strongly disagree) to 7 (strongly agree). To detect the number of components of the self-report instrument, a principal component analysis was conducted with orthogonal rotation (varimax). Examination of the scree plot and eigenvalues over 1 suggested the presence of one component that accounted for 71.24% of the variance. The scale was reliable, Cronbach’s alpha = .93. A mean score was calculated, with higher scores representing higher experiences of PRD. See Table A in S1 Appendix for the items.

#### Assessment of game experience

It was additionally explored whether participants’ liking of the game (i.e. how much fun the card game was; if they would like to play this game again) and their involvement (i.e. if they cared about the points earned; if they did their best; if they cared about the points their opponent earned). The answer scale ranged from 1 (strongly disagree) to 7 (strongly agree). An average score of the liking items was calculated (Cronbach’s alpha = .89), and the involvement items were analysed separately, as this scale was not reliable (Cronbach’s alpha = .56)

### Results

A t-test showed that the PRD condition (*M* = 5.33, *SD* = 1.33) scored significantly higher than the control condition on the experienced PRD scale (*M* = 2.37, *SD* = 1.03), *t* (170) = 16.29, *p <* .001, 95% CI [2.61–3.33], *d* = 2.49. Conditions did not differ significantly in game liking, *t*(170) = 1.60 *p* = .11. Conditions did not differ either in caring about their points, *t*(170) = 1.21 *p* = .23, or their opponent’s points, *t*(170) = 1.22 *p* = .23, or doing their best, *t*(170) = 0.45 *p* = .65. On average, participants rated a positive liking experience (M = 5.33, *SD* = 1.37) and reported that they cared about their points (M = 5.58, *SD* = 1.46) and their opponent’s points (M = 4.88, *SD* = 1.80) and that they did their best (M = 5.33, *SD* = 1.37). See Table B in S1 Appendix for means and SDs of items per condition.

### Discussion

This manipulation was considered successful, as conditions differed significantly in their PRD experience during the card game. Furthermore, both conditions reported liking the game and caring about earning points (i.e. the main goal of the game). This is relevant, because it ensures that potential differences in food choice are not the result of plausible side-effect game experiences. Moreover, these observations indicate that the outcome was indeed desired, an important precondition of experiencing relative deprivation [16,19].

## Study 2: Effect of PRD on hypothetical food choice

The aim of Study 2 was to test the effect of PRD on (hypothetical) food choice. The points earned in the card game served as resources to select (purchase) food products in an online food shopping task. It was hypothesized that the PRD condition would select more palatable, snack-type (i.e. rewarding) food products than the control condition. Hypotheses and methods were preregistered on Open Science Framework prior to data collection (https://osf.io/vk6mq).

### Methods

#### Participants

Participants aged 18–70 years who were fluent in English were recruited via Prolific and were compensated with £1.30 upon completion of the study (average completion time was 13 minutes). Other inclusion criteria were no prior participation in Study 1 and no allergy for gluten, dairy/lactose, eggs, nuts, and wheat/grain. Based on a power calculation using G*Power (alpha of .05, power of .90, effect size (Cohen’s *f*) of .2) and an additional 10% of oversampling allowing for exclusion, the sample size for recruitment was 292. A small to medium effect size was expected, given that food choice is a multifactorially determined behaviour and plausibly also/most affected by individual differences in liking of the specific products [47,48]. Six participants were excluded from analysis based on *a priori* exclusion criteria (i.e. incorrect answers on both attention check items (N = 2) and an exceptional completion time of below 6 minutes (*N* = 1) or higher than 35 minutes (*N* = 3), based on Study 1, suggesting inadequate performance). As a result, the sample for analysis consisted of 287 participants (58.2% male, 41.1% female, 0.01% other) with a mean age of 30.57 years (*SD* = 10.52, range 18–68). Of the 44 participating nationalities, the most frequent were British (19.2%), American (11.5%), and Polish (11.1%).

#### Procedure

After providing written informed consent, participants completed the Revised PRD Scale [21] and the Power of Food Scale [49] (see description of these control measures below). Next, they were randomly assigned to either the PRD or the control condition and played the card game described in Study 1 (PRD manipulation) in which they earned points that served as resources to be spent in the succeeding online food shopping task. Participants were then asked about their level of hunger, weight in kg, and height in cm (conversion information was provided, i.e. 1 inch = 2.54 cm and 1 pound = 0.4536 kg), dietary concerns, and demographic information including gender, age, nationality, and last completed education. Lastly, participants were thanked and debriefed. The study was approved by The Social Science Ethics Committee of Wageningen University.

#### Online food shopping task

Participants were asked to choose from eight products presented on screen, using their total earnings of 30 points. Each product picture portrayed one serving for immediate consumption. Four typical high-sugar/fat snack-type food products (i.e. chocolate cookie, chocolate bar, two types of crisps) were deemed as ‘rewarding’ foods and the other four products (i.e. unsalted peanuts, muesli bar, rice waffles, pear) were deemed as ‘neutral and healthy’. This classification was determined based on a pilot study in which participants rated the palatability and healthiness of food products (see S2 Appendix for details). As each product ‘cost’ 10 points, participants had to select three products (different products or more of the same product). This choice was obviously hypothetical, i.e. participants did not receive chosen products. To approach a sense of ‘wanting’, i.e. the anticipation of pleasure due to learned associations with reward [31], participants were instructed to imagine that the foods were immediately available to them and to base their food choice on what they desired the most at that moment. The dependent variable was the number of rewarding food choices, ranging from 0 to 3.

#### Control variables

*Chronic PRD*. Chronic tendencies for PRD were measured, because it was reasoned that these may in themselves influence food choices [44] and interfere with the state of experiences of PRD. The 5-item revised Personal Relative Deprivation Scale [21] was used, which assesses the extent to which participants feel subjectively worse off compared with others (e.g. “I feel deprived when I think about what I have compared to what other people like me have”) on a 6-point Likert scale ranging from 1 (strongly disagree) to 6 (strongly agree). A mean score was calculated for the 5 items. The scale had a Cronbach’s alpha of .76.

*Sensitivity to palatable food*. The Power of Food Scale (Cronbach’s alpha = .93) measures appetite for palatable foods and the psychological influence that the food environment has on the individual [49]. The scale has 15 items (e.g. “I find myself thinking about food even when I’m not physically hungry”) answered on a 5-point scale ranging from 1 (I don’t agree at all) to 5 (I strongly agree). A mean score was calculated. Sensitivity to palatable food was included because this trait-level factor may obviously have considerable influence on rewarding food choices in this experiment [50,51].

*Hunger*. Participants were asked how hungry they were on a 7-point scale ranging from 1 (not at all) to 7 (very much), as this has been shown to be a primary motive for eating [52] and may also influence food choices.

*BMI*. Body mass index was calculated by dividing self-reported weight (kg) by the square of the person’s height (m^2^). Seven participants had an unrealistic BMI value (> 271) because of a low value for height (< 1 metre); these were coded as missing values. BMI was included because it has been reported that people with overweight or obesity tend to choose smaller immediate rewards [53,54], which may translate into more rewarding food choices.

*Dietary concern*. Dietary restraint was measured with the 6-item Concern for Dieting subscale of the Revised Restraint Scale, which assesses attitude towards dieting [55]. Items were answered on a 4 to 5-point scale. The scale had a Cronbach’s alpha of .72. Dietary concern was included because dieters’ food choice may be predominantly based on weight-control strategies such as eating fewer calories [56].

### Results

#### Descriptives, correlations, and comparability between conditions

Following the preregistration, an analysis of significant correlations between the control variables and the dependent variable–rewarding food choice–resulted in the identification of sensitivity to palatable food and hunger as covariates. T-tests revealed that conditions did not differ on chronic PRD, sensitivity to palatable food, hunger, age, BMI, and dietary concern, *t* < 1.11, *p* > .269. Conditions did not differ either on gender, *χ*^*2*^ (2, N = 287) = 2.16, *p* = 0.34, nationality, *χ*^*2*^ (43, N = 287) = 45.02, *p* = 0.39, or education level *χ*^*2*^ (8, N = 287) = 8.79, *p* = 0.36, suggesting that randomization was successful. See Table C in S1 Appendix for the correlations between variables and the means and standard deviations (SDs) per experimental condition.

#### Test of hypothesis

Neither of the identified covariates (i.e. hunger and sensitivity to palatable food) interacted with condition for rewarding food choice, meaning that the assumption of homogeneity of regression slopes was met. As the residuals of the mean number of rewarding food choices were non-normally distributed, bootstrapping (10,000 samples) was applied [57]. There was no significant main effect of experimental condition on the mean number of rewarding food choices, *F*(1, 283) = 1.08, *p* = .60, 95% CI[-0.29, 0.17], *η*_*p*_^*2*^ = 0.001. Participants in the PRD condition (*M*_adj,_ = 1.73, *SE* = 0.08) did not differ from those in the control condition on the number of rewarding foods chosen (*M*_adj,_ = 1.67, *SE* = 0.08). The covariates hunger and sensitivity to palatable food were significantly related to rewarding food choice, *p*’s < .05. Tests of hypotheses and exploratory analyses were also performed without serious outliers in BMI (outliers were identified according to a Z-score criterion of ± 3,i.e. BMI > 56)). Exclusion of these seven outliers did not change the results.

#### Exploratory analyses, preregistered

**Fig 2:**
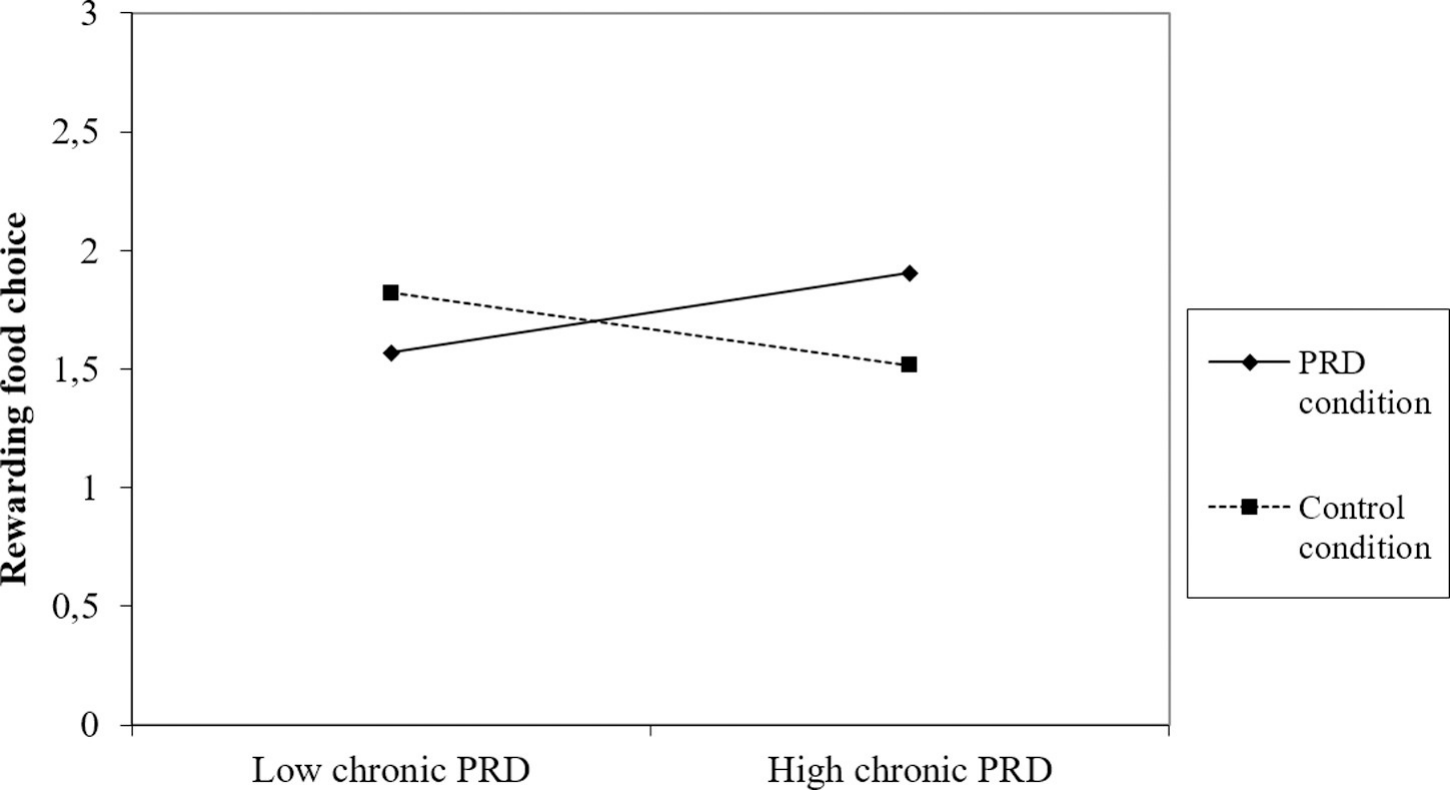
Interaction effect observed in Study 2.

Exploratory analyses of two-way interactions between experimental condition and control variables on rewarding food choice were conducted, using the PROCESS macro for a bootstrapped test [58]. A significant disordinal interaction between condition (centered) and chronic PRD (centered) on rewarding food choice was found, *F*(1, 283) = 7.08, *p* = .008, R^*2*^ *-*change = 0.02, also when the identified covariates were controlled for, *F*(1, 281) = 4.48, *p =* 0.04, R^*2*^ *-*change = 0.01. See Fig 2 for a visualisation of the interaction effect. Simple effects including identified covariates demonstrated that conditions did not differ significantly on rewarding food choice for participants with a high level of chronic PRD (+1SD; 4.02), B = .30, *t*(281) = 1.71, *p* = .087, 95% CI [-0.64, 0.04], average level of chronic PRD (3.09), B = .06, *t*(281) = .53, *p* = .59, 95% CI [-.29, .18], or a low level of chronic PRD (-1SD; 2.16), B = -.17, *t*(281) = -1.19, *p* = .23, 95% CI [-0.11, 0.47]. Simple effects without covariates showed the same pattern of results as those with covariates, except that the simple effect of high level of chronic PRD was significant (B = -.38, *t*(283) = -2.16, *p* = .03, 95% CI [-0.72, -0.03]). This difference was in the hypothesized direction, indicating that the PRD condition chose more rewarding foods than the control condition under high levels of chronic PRD. No interaction effects were found between condition and any other control variables, *p* > .21.

### Discussion

No main effect of the PRD manipulation on food choice was found. However, the manipulation seemed to have a differential effect for different levels of chronic PRD. Simple effects bordered on significance, showing a pattern that those that were relatively deprived in the card game and also experienced higher chronic relative deprivation appeared to select a higher number of rewarding foods. It may be reasoned that individuals already feeling deprived were more sensitive to the manipulation with respect to its influence on food choice, and that those who did not experience PRD in daily life did not translate incidental feelings of PRD into hypothetical food selection. A limitation of this study was that food choices were hypothetical; therefore, the experiment was next conducted in a lab-in-the-field setting.

## Study 3—Effect of PRD on non-hypothetical (real) food choices

The aim of Study 3 was to conceptually replicate the online study (Study 2) in a lab-in-the-field setting in which participants from a community sample made real, non-hypothetical food choices. It was hypothesized that participants in the PRD condition would choose more palatable (rewarding) snack-type food products than participants in the control condition (i.e. a main effect). Moreover, given the results of the online study, this effect could be expected to be observed only, or at least to a greater extent, in participants scoring high on chronic PRD. The aim, hypothesis, and methods were preregistered on Open Science Framework prior to data collection (https://osf.io/fwy8r).

### Participants

A total of 308 women were recruited at a one-week summer fair (*Libelle Zomerweek*), where they could participate in our workshop “grocery shopping game”. Twenty-one rounds of the workshop were held across the fair (at 10.30, 11.30, 12.30, 14.30, 15.30, and 16.30 h) in maximum groups of 15 participants. Given these constraints, the maximum sample size that could be reached was 315 participants. Participants could enroll for the workshop at the central registration desk and, to fill the open spots, some were actively recruited by research assistants. Twenty-five participants were excluded from analyses because they had an allergy or intolerance for gluten, dairy/lactose, eggs, nuts, soja, or wheat/grain, or followed a vegan diet. Additionally, 19 participants were excluded because they did not adhere to instructions in one or multiple ways (i.e. taking more or fewer than three products, stopping before the food choice task, performing the task together with a friend). Four participants that correctly guessed the purpose of the study were also excluded. Hence, the analytic sample consisted of 260 participants, with an average age of 48.75 (*SD* = 14.05 range 16–76) and various educational backgrounds (categorized into 18.8% low, 42.2% middle, 37.3% high, according to a classification of Statistics Netherlands (www.cbs.nl), and 0.8% indicated ‘other’). A post-hoc power calculation using G*Power indicated that with this sample size, a power of .80 could be reached for a small to medium effect size of the hypothesized main effect, Cohen’s *f* = .175.

Participation in this annual summer fair was considered a good opportunity for efficient recruitment of a large and diverse community sample. As this female sample differed from the mixed gender sample in Study 2, we checked whether the results of Study 2 differed for men and women: no two-way interaction between gender and condition was found, and no three-way interaction between gender, chronic PRD, and condition was found.

### Procedure and measures

Participants were seated at a long table and were separated by shields so that they would complete the study individually. Each participant was provided with a participant number, a handout with the steps, and a tablet computer. At the start of the workshop, participants were collectively introduced to the study “about grocery shopping” and were instructed not to talk with one another. The same methodology (i.e. informed consent, manipulation, and measures) as in the online study was applied, with the following adaptations. First, for feasibility reasons, the procedure was shortened by excluding the nationality measure and by using the 4-item Present Food subscale of the Power of Food Scale [49] and one item from the Dietary Concern scale, i.e. “How conscious are you of what you are eating?” [55]. The 4-item Present Food subscale of the Power of Food Scale involves “reactions to palatable foods when they are physically present but have not yet been tasted”. This subscale (Cronbach’s alpha = .84 in the current study) was conceptually closest to food choice and correlated most strongly with food preference in the online study. The 1-item dietary concern was based on a principal component analysis with varimax rotation of the online study data, which suggested two components; one of the components containing this item (loading .92) was conceptually closest to food choice. Also, weight and height items were deleted to avoid participants feeling uncomfortable answering these items. Second, the food choice measure was realized by letting each participant collect products from a wooden crate positioned at her table (see *Food choice task*). Third, in the instructions for the card game, it was already explained that each product cost 10 points, as it was arguably better to know beforehand the value of the points that could be earned. Fourth, a final question asked what participants thought the purpose of the study was. Participants were collectively debriefed via a quiz. The study was approved by The Social Science Ethics Committee of Wageningen University. Prior to this study, a non-preregistered pilot study was conducted to test the feasibility of the PRD manipulation and a different form of the food shopping task among a community sample (*N* = 101, see S4 Appendix).

### Food choice task

**Fig 3:**
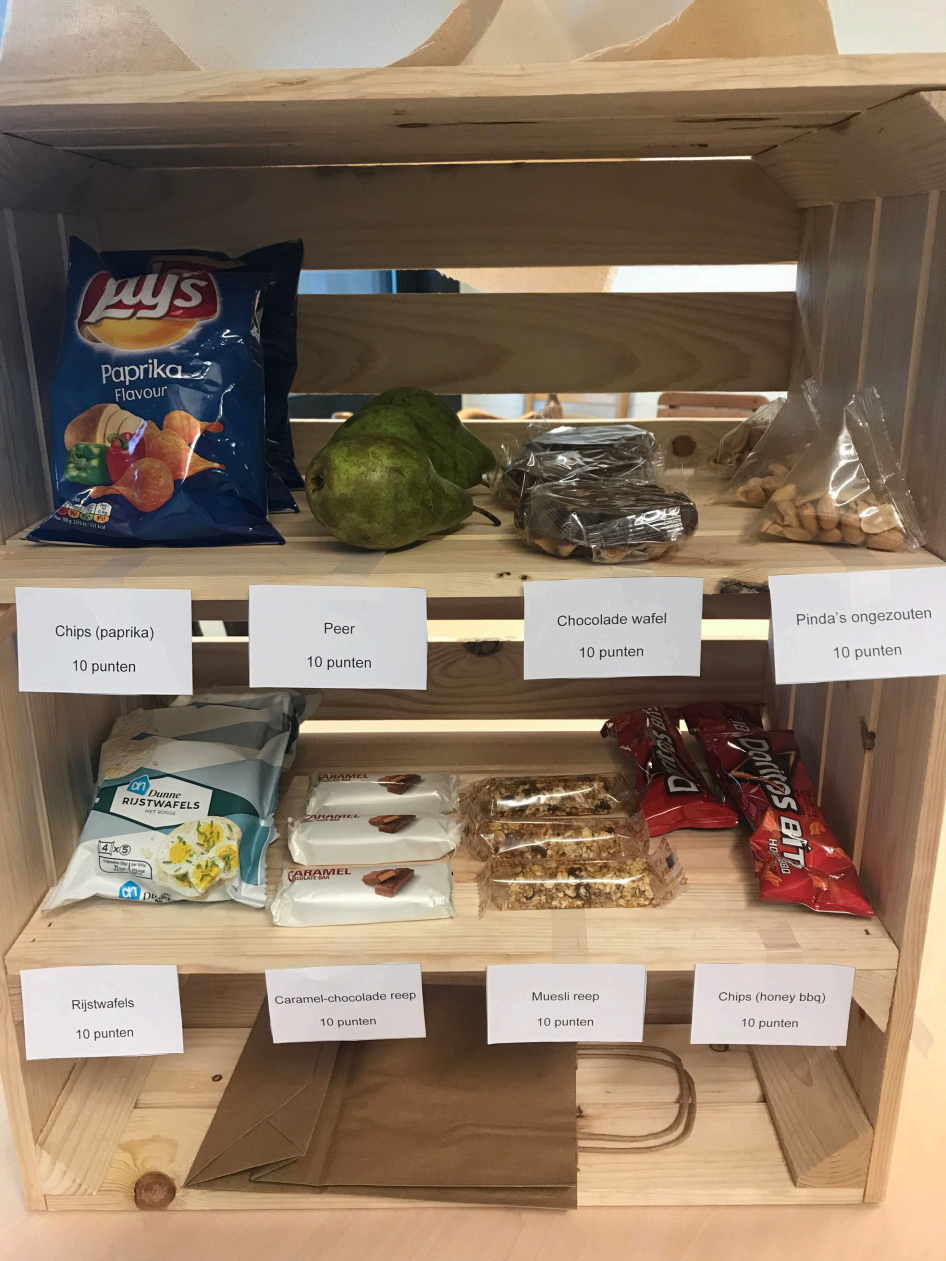
Picture of the food choice crate in Study 3.

Participants were instructed to spend their points earned in the card game on groceries. Each product cost 10 points, as indicated on Qualtrics and on the ‘price tag’ near each product. Participants chose products from a cloth-covered crate containing two shelves that presented the eight different products (four products per shelf, three pieces of each product). The placement of the products was identical for all participants (see also Fig 3). Participants were instructed to remove the cloth from the crate and to put their choice of products in a paper bag. On the Qualtrics instruction screen, it was stated that participants should not think for too long and choose something that they would want to eat now. In conformity with the online study, the rewarding foods were chocolate waffle, chocolate bar, and two types of crisps. The neutral, healthy foods were muesli bar, pear, rice waffles, and unsalted peanuts. All foods were served as single-portion packages (except the pear). After participants left, food choices were recorded by the researchers (who were blind to the condition assignment) by counting the products taken.

### Results

#### Descriptives, correlations, and comparability between conditions

An analysis of significant correlations between the control variables and the dependent variable–rewarding food choice–resulted in the identification of age, education level, dietary concern, and sensitivity to palatable food (Present Food subscale) as covariates. T-tests showed that conditions did not differ on the control variables sensitivity to palatable food (Present Food subscale), hunger, age, and dietary concern, t(258) < 1.72, p > .09 and education, *χ*^*2*^ (7, N = 258) = 11.58, *p* = .12. Table D in S1 Appendix presents the correlations between the variables under study and the means and standard deviations (SDs) per condition.

#### Test of hypotheses

None of the identified covariates (i.e. age, education level, dietary concern, and sensitivity to palatable food) interacted with condition for rewarding food choice, meaning that the assumption of homogeneity of regression slopes was met. A bootstrapped (10,000 samples) ANCOVA showed that there was a significant main effect of experimental condition on the mean number of rewarding food choices, *F*(1, 250) = 4.61, *p* = .031, 95% CI[0.03, 0.45], *η*_*p*_^*2*^ = 0.02. Participants in the PRD condition (*M*_adj,_ = 1.56, *SE* = 0.16) chose more rewarding foods than those in the control condition (*M*_adj,_ = 1.33, *SE* = 0.17). The covariates education level, dietary concern, and sensitivity to palatable food were significantly related to rewarding food choice, *p*’s < .01. Age was not significantly related to food choice, *p* = .14. This main effect was not significant without any covariates, F(1, 258) = 2.49, p = .11, 95% CI[-.0.04, 0.40], *η*_*p*_^*2*^ = 0.01. Controlling only for sensitivity to palatable food resulted in a significant main effect, F(1, 257) = 3.97, p = .043, 95% CI[0.01, 0.44], ηp2 = 0.02, indicating that this was an important covariate in the testing of hypotheses.

To analyse whether condition affects food choice differently for scores on chronic PRD (in conformity with Study 2), it was tested whether chronic PRD (centered PRD scale) interacted with experimental condition (centered), using bootstrapping in PROCESS [58]. This interaction was not significant, *F*(1, 256) = 1.86, *p* = .17, *R*^*2*^-change = 0.01; nor was it significant when the identified covariates were controlled for *F*(1, 248) = 0.53, *p* = .47, *R*^*2*^-change = 0.00. Removing two participants with the highest chronic PRD scores (> 4.20) from the sample did not change these non-significant results of this two-way interaction, ANOVA model *p* = .07, ANCOVA model, *p* = .37.

#### Exploratory analyses, preregistered

Three-way interactions between experimental condition, chronic PRD, and each of the control variables were explored with PROCESS [58]. A significant interaction was found between centered condition, centered chronic PRD, and centered age, *F*(1, 252) = 4.32, *p* = .039, 95% CI[0.001, 0.048], *R*^*2*^-change = .02, also when the identified covariates were controlled for, *F*(1, 245) = 5.03, *p* = .026, *R*^*2*^-change = 0.02. This adjusted three-way interaction was further disentangled by bootstrapped conditional effects. No significant conditional effects of the two-way interaction between chronic PRD and condition on rewarding food choice were found for participants with higher age (+ 1SD; 62.8 years), *B =* .45, *t*(245) = 1.97, *p* = .050, 95% CI [0.001, 0.89], lower age (- 1 SD; 34.7 years), *B =* -.22, *t*(245) = -1.91, *p* = .23, 95% CI [-0.58, 0.15], or medium age (48.8 years) *B =* .11, *t*(245) = 0.79, *p* = .43, 95% CI [-0.001, 0.41]. As the answer items of the PRD scale ranged from 1 to 6, it appears that PRD scores were overall rather low; these low, medium, and high ranges need to be interpreted in relative terms. Other three-way interactions between condition, chronic PRD, and any control variable other than age were not significant, all *p*’s > .15.

#### Discussion

On average, participants in the PRD condition chose more rewarding foods than those in the control condition, particularly when sensitivity to palatable food was controlled for. An interaction between condition and chronic PRD, as observed in Study 2, bordered on significance for participants in the higher age group.

## General discussion

Drawing on the relative deprivation hypothesis and the theory of justice motivation [23], the aim of this research was to demonstrate the effect of experiences of PRD on food choice behaviour. In two preregistered experimental studies, some preliminary evidence was found for an effect of induced PRD on hypothetical and real food choices in a grocery shopping setting. Across the studies, a difference in food choice between conditions bordered on significance and hence the findings need to be interpreted with caution. The effect of PRD on real, non-hypothetical food choice appeared significant, when trait-level sensitivity to palatable foods was controlled for. A numerical difference was repeatedly in the hypothesized direction: those who were unfairly relatively deprived of resources earned in a card game spent these resources on more palatable, energy-rich food products (rather than neutral tasting, healthier foods), although this effect may be truer for those with higher chronic PRD.

This is one of the first studies to demonstrate some causal effect of feelings of PRD on actual food choice behaviour. Building on previous studies showing that chronic-level PRD is associated with health outcomes [19,20] and state-level PRD with an inclination towards immediate rewards [21], our results show that PRD may result in more palatable and unhealthy food choices. These findings are relevant in light of the current obesogenic environment characterized by its abundance of unhealthy foods, where it appears that part of society is disproportionally affected by these temptations. A large body of evidence indicates that inequality and obesity and health are linked at the societal level. The (preliminary) results of this study suggest that this association may be partly due to the effect of relative deprivation (a downstream psychological consequence of inequality) and food choices at the individual level.

Specifically, we carefully manipulated relative deprivation on the basis of a theoretical conceptualisation of PRD, including both cognitive (‘being worse-off’, ‘being unfairly treated’) and affective responses (anger, resentment) to unequal/unfair outcomes, stemming from upward comparisons [15,16]. Notably, as all participants in this study received objectively the same number of resources, this research indicates the relevance of targeting the perception of inequality and unfairness beyond a person’s absolute, economic situation. Indeed, Inglis, Ball [59] showed that socioeconomic inequalities in the healthfulness of food choices were not reduced through varying food budgets available to women with low and high incomes. Decreasing economic inequality has been declared as the approach to improve a nation’s health [60], but, with respect to dietary behaviours, this may be insufficient when not accompanied by a reduction in experiences of injustice stemming from social comparisons.

Our findings have important implications for theorizing about food choice behaviour under states of deprivation in two main ways. First, the finding that those who were relatively deprived chose more snack-type foods that were assessed as relatively unhealthy and highly palatable may imply that the hedonic–and so immediately rewarding–properties of foods become more important under deprivation. Hence, an implicit assumption for this reasoning is that the act of making food choices under deprivation theoretically entails a subconscious motivational trade-off between immediate pleasure and long-term health goals; this accords with empirical evidence for the relation between PRD and a preference for immediate rewards [21,22] and with research on self-regulation of eating [61]. However, in previous studies on SSS and caloric preference/intake, such a trade-off categorization has not been made and findings have generally been explained with an evolutionary approach that assumes that people have a functional, adaptive motivation to compensate for resource scarcity directly with calorie-rich foods, as this promotes survival [37,38,62]. This functional explanation could apply to the present study as well, especially given a sub-analysis showing that PRD also affected actual food choice when calorie-rich peanuts, which were not perceived as particularly tasty, were additionally categorized as rewarding foods rather than as neutral, healthy foods. Hence, it remains questionable whether PRD leads to a particular preference for energy versus hedonic properties of foods, although these specific processes may be hard to disentangle as the reward system has evolved such that humans prefer energy-dense sweet and fat foods [63,64]. More generally though, individuals’ liking of foods evolves over the life course as a result of socio-cultural influences, including food exposure [65,66]. It remains an avenue for future research on socioeconomic disparities in diet quality to investigate how the food liking of people on various SES levels is shaped by various socio-economic/cultural factors, including experiences of deprivation and differential exposure to food availability.

Second, the finding that the effect of state-level PRD on rewarding food choice was moderated by higher chronic feelings of PRD may elucidate the challenge of recapitulating an intrusive level of PRD as experienced by individuals actually living under deprived conditions [35]. Particularly, a plausible explanation for this finding is that only those with chronic PRD actively engaged in the self-comparison that is needed for a true PRD effect. Moreover, these findings may contribute to insights into how food choice behaviour is formed by previous experiences of deprivation. In light of studies focused on emotions as a stimulus for the conditioning of food craving and selection [67,68], our findings may imply that state-related deprivation may lead to palatable food selection because of a learned response to feelings of PRD, reflecting a form of habitual reinforcement [69] or more generally, of poor emotion regulation. Notably, a series of studies on the influence of childhood SES on food intake in a laboratory setting, showed that for those raised in high SES environments, food intake varied according to physiological energy need, but for those raised in low SES environments, food intake was high independent of of current energy needs [70]. It was theorized that early exposure to low SES may become embedded in energy regulation systems in later life, plausibly due to learning experiences with deprivation. Further research is needed to further disentangle whether and how states of PRD and childhood or chronic experiences of PRD may interact in the influence on food preferences/cravings and dietary patterns.

This study has some particular strengths. First, the experiment with real, non-hypothetical food choices was conducted in a community sample with various educational backgrounds, rather than in a homogenous highly educated student sample as commonly used in previous studies on social status and eating [27,38,40,44,71]. Second, we employed a real-time induction of PRD experiences where participants were actually unfairly deprived of resources compared to a (fictitious) opponent player, and we believe that this method is more powerful than a person imagining him/herself in a hypothetical scenario. Moreover, the resources that were the source of relative deprivation were directly used in the grocery shopping task, and this enhances ecological validity.

Yet, there may be some limitations regarding the conceptualisation, operationalisation and measurement of personal relative deprivation that we employed. First, the applied conceptualisation of relative deprivation was rather comprehensive, not only including the act of upward social comparisons, but also perceptions of unfairness and negative emotions. According to theories of relative deprivation [14–17], a sense of unfairness is part of PRD because human justice concern is a precondition for the experience of relative deprivation–the sense that one does not get what he/she deserves relative to others. Interestingly, a series of experiments testing social comparison processes in PRD showed that feelings of unfairness mediated the effect of adverse social comparison on feelings of resentment and dissatisfaction with a financial outcome [72]. Yet, in the wider context of aiming to understand the link between societal inequality and unhealthy diets, it may appear questionable whether feelings of unfairness is a relevant additional process as compared to the mere upward social comparison process. Previous studies have shown that upward social comparisons without feelings of unfairness per se influenced unhealthy eating behaviour [35,36]. For example, people that perceived to have a lower relative income than their interaction partners consumed more calories [27]. Hence, it would be of interest for future research to test whether and how a mere upward social comparison process would produce similar effects on eating behaviour as compared to feelings of being unfairly disadvantaged.

Second, one may question the validity of our self-developed digital manipulation with respect to the social comparison process. Participants were led to believe that they played against a previous participant with the same age and gender, and they were explicitly presented with the relative earnings, but it remains questionable to what extent they truly compared themselves with a similar other, rather than a computer or a dissimilar other. Although the manipulation check demonstrated that the PRD manipulation indeed led to feelings of being worse-off, the check items included wording specifying the comparative nature of the feelings. Hence, it cannot be verified to what extent the manipulation distinguishes from general forms of negative affect that are not due to social comparison per se. However, the digital design of the PRD manipulation provided great benefits in terms of feasibility as well as reliability, as it allowed us to compose a uniform game that included the exact same amount of relative deprivation ‘portions’ across all participants in the PRD condition. Building on the aforementioned strength of inducing real-time deprivation of resources that were actually spent on food products, we encourage future experimental studies on effects of PRD to also involve real persons (e.g. research confederates) for a more optimal social comparison process.

This study has also some methodological limitations regarding the assessment of food choice. First, the food products offered during the grocery shopping task were limited in variety and may not represent the foods chosen by the participants in daily life. Nevertheless, we selected products with which most people are familiar, and in sub-analyses we observed that all products were substantially selected by the samples, allowing some variety in popularity. Second, we assessed food preference at only one juncture, and more long-term assessments of food purchases are necessary to generalize to dietary patterns. Third, all foods had the same ‘price’ in the food shopping task, whereas in real life these foods have different prices. Nevertheless, we counterbalanced for actual prices of products in the two categories of foods (i.e. rewarding versus neutral) so that these categories were equally expensive. Future studies on the effect of PRD on food choice may disentangle the relative importance of price and palatability of the presented foods. Fourth, presented foods were categorized into rewarding versus neutral foods based on perceived palatability and healthiness. Other, unmeasured food properties (e.g. convenience of consumption) may also have differed between these categories and may have confounded the outcome. To investigate the idea that PRD leads to a heightened sensitivity to rewarding foods, we suggest that future studies should focus on a more proxy measure of rewarding food preference, such as rewarding value of food [51,73] or food cue reactivity [74]. Fifth, in Study 3, the sample consisted of women only and so its findings cannot be generalized to men. Yet, studying a female population may be especially relevant, as the social status and BMI relationship has been more consistently found among women than among men [35,36,75]. Future research on PRD and dietary outcomes may need to identify any differential effects for gender. Sixth, the perceived palatability and healthfulness of the foods was not consistently evaluated in each experiment. Although the selection of foods was based on previous studies [76–78], the lack of these data in our series of studies precludes evaluation of the extent to which null effects were the result of invalid food categorization across our different samples and/or within individuals. This is of particular relevance for Study 3 in which we recruited a female sample and measured real food choice. Furthermore, apart from feasibility concerns (i.e. time constraints), the validity of these evaluations pre or post experiment may be questionable, given potential carryover effects.

Finally, in neither Study 2 (hypothetical food choice) nor Study 3 (actual food choice), participants consumed the foods selected, which may be considered a limitation given that the hedonic experience is directly associated with reward. However, according to Kringelbach, Stein and van Hartevelt [79], the food pleasure cycle consists of three stages: anticipation/wanting, consumption/liking, and learning (our food choice measurement most likely tapping in the first stage, building on Pavlovian associations). In both our studies we included well-known food products, and we instructed participants to base their choice on what they would like to eat at that moment. Based on neuroimaging studies [34], it was reasoned the (palatable) food products shown on screen (study 1), and especially the products that were actually available for immediate or later consumption (study 2) would elicit reward responding related to ‘wanting’, which is an important driver of reward seeking behaviours including food choice [31,80].

Although conclusions can only be drawn with reservation, this research found some initial evidence for a causal effect of experiences of relative deprivation on unhealthy food choices. Effect sizes of this multifactorial behavioural outcome were small, and more research is needed to establish more firmly a causal link between relative deprivation and unhealthy dietary behaviours. Nevertheless, this research provides initial support for the idea that targeting inequality and injustice, and their psychological consequences, may be part of the solution to reduce societal disparities in the healthfulness of dietary patterns.

## Supporting information

S1 Appendix Supplementary tables. (DOCX)

S2 Appendix Pilot study: Food rating. (DOCX)

S3 Appendix Results “bogus” task Study 1. (DOCX)

S4 Appendix Pilot study: Testing PRD manipulation and non-hypothetical food choice task. (DOCX)
