# Supplementary material for: The effect of personal relative deprivation on food choice: An experimental approach
Source: PLoS One. 2022 Jan 13;17(1):e0261317. doi: 10.1371/journal.pone.0261317 (PMC8758004; doi:10.1371/journal.pone.0261317)
Supplement: S1 Appendix — (DOCX) [file pone.0261317.s001.docx]

**S1 Appendix.**

**Supplementary Tables.**

**S1 Table A.** Items of the Experienced PRD scale that functioned as manipulation check of the card game

| 1. I felt worse-off when I compared myself with my opponent |
| --- |
| 2. I think the amount of points I earned compared to my opponent was unfair |
| 3. I felt that the amount of points I earned compared to my opponent was just^a^ |
| 4. I felt I was deprived when I compared my points with my opponent |
| 5. I felt resentment when I saw how many points I earned compared to my opponent |
| 6. I felt satisfied with the amount of points I earned compared to my opponent^a^ |
| 7. I was frustrated when I saw how many points I earned compared to my opponent |

^a^reverse coded

|  | | Mean (SD) | |
| --- | --- | --- | --- |
|  | | PRD condition | Control condition |
| The card game was fun to play |  | 5.28 (1.43) | 5.64 (1.25) |
| I would like to play this card game again |  | 5.05 (1.56) | 5.35 (1.48) |
| I cared about the points I earned |  | 5.45 (1.58) | 5.72 (1.32) |
| I did my best during the card game |  | 6.44 (.85) | 6.49 (.81) |
| I cared about the points my opponent earned |  | 4.71 (1.93) | 5.05 (1.65) |

**S1 Table B**. Means and SDs of items of game liking and involvement per condition

Scored on 7-point scale

**S1 Table C.** Means, SDs, and correlations (Spearman’s rho) of the variables in Study 2

|  | 1 | 2 | 3 | 4 | 5 | 6 | 7 | 8 | 9 |
| --- | --- | --- | --- | --- | --- | --- | --- | --- | --- |
| 1. Rewarding food choice^a^ | - |  |  |  |  |  |  |  |  |
| 2. Chronic PRD | .01 | - |  |  |  |  |  |  |  |
| 3. Sensitivity to palatable food | .20** | .07 | - |  |  |  |  |  |  |
| 4. Hunger | .19** | -.00 | .35** | - |  |  |  |  |  |
| 5. BMI | .01 | .01 | .19** | -.01 | - |  |  |  |  |
| 6. Dietary concern | .04 | .07 | .54** | .16** | .28** | - |  |  |  |
| 7. Age | .01 | -.10 | -.06 | -.08 | .19** | -.00 | - |  |  |
| 8. Gender^a^ | .04 | -.00 | .28** | .04 | -.09 | .25** | .17** | - |  |
| 9. Education level | -.10 | -.09 | -.05 | -.05 | .12 | .11 | .26** | .03 | - |
| Mean PRD (*N* = 145) | 1.74 | 3.08 | 2.88 | 3.81 | 26.19 | 13.88 | 30.60 | 56.6^a^ |  |
| SD | .99 | .91 | .91 | 1.65 | 8.37 | 30.50 | 10.32 |  |  |
| Mean control (*N* = 142) | 1.66 | 3.11 | 2.90 | 3.58 | 26.43 | 14.32 | 30.55 | 59.9^a^ |  |
| SD | 1.02 | .96 | .92 | 1.75 | 8.70 | 3.72 | 10.75 |  |  |
| Mean overall | 1.70 | 3.09 | 2.89 | 3.70 | 26.31 | 14.10 | 30.57 | 58.2^a^ |  |
| SD | 1.00 | .93 | .91 | 1.70 | 8.52 | 3.61 | 10.52 |  |  |

** correlation significant at *p* < .01. ^a^ percentage males.

**S1 Table D.** Means, SDs, and correlations (Spearman’s rho) of the variables in Study 3

|  | 1 | 2 | 3 | 4 | 5 | 6 | 7 |
| --- | --- | --- | --- | --- | --- | --- | --- |
| 1. Rewarding food choice | - |  |  |  |  |  |  |
| 2. Chronic PRD | .06 | - |  |  |  |  |  |
| 3. Sensitivity to palatable food^a^ | .25*** | .04 | - |  |  |  |  |
| 4. Hunger | .07 | -.04 | .17** | - |  |  |  |
| 5. Dietary concern^b^ | -.25*** | -.05 | -.20**. | -.11 | - |  |  |
| 6. Age | -.14* | -.07 | -.27*** | -.05 | .05 | - |  |
| 7. Education level^b,c^ | -.18** | -.05 | .08 | -.06 | .15* | -.25*** | - |
| *Mean* PRD condition (*N* = 134 ) | 1.51 | 1.94 | 3.00 | 2.82 | 3.62 | 13.19 | *NA* |
| *SD* | .99 | 0.79 | 1.02 | 1.61 | .94 | 1.14 | *NA* |
| *Mean* control condition (*N* = 126) | 1.33 | 1.93 | 3.18 | 3.17 | 3.64 | 14.82 | *NA* |
| *SD* | .87 | 0.75 | .94 | 1.64 | .95 | 1.32 | *NA* |
| *Mean* overall | 1.42 | 1.93 | 3.09 | 2.99 | 3.63 | 48.75 | *NA* |
| *SD* | .93 | .77 | .98 | 1.63 | .94 | 14.05 | *NA* |

* correlation significant at *p* < .05 ** correlation significant at *p* < .01. *** correlation significant
at *p* < .001. *NA* = not applicable. ^a^ Present Food subscale of Power of Food Scale (1).

^b^ two missing values. ^c^ Recoded from 9 categories to 4: low, middle, high, and other.

**S1 references**

1. Lowe MR, Butryn ML, Didie ER, Annunziato RA, Thomas JG, Crerand CE, et al. The Power of Food Scale. A new measure of the psychological influence of the food environment. *Appetite*. 2009;53(1):114-8.
