# Supplementary material for: The effect of personal relative deprivation on food choice: An experimental approach
Source: PLoS One. 2022 Jan 13;17(1):e0261317. doi: 10.1371/journal.pone.0261317 (PMC8758004; doi:10.1371/journal.pone.0261317)
Supplement: S2 Appendix — (DOCX) [file pone.0261317.s002.docx]

**S2 Appendix.**

**Pilot study: Food rating**

A clear distinction in palatability and healthiness was warranted to induce the choice more as a trade-off reflecting the motivation to enjoy palatable foods versus the motivation to adhere to long-term health goals. Therefore, products presented in the food shopping task had to include an equal amount of relatively palatable but unhealthy foods products as well as relatively healthy foods that were less palatable. Our selection of products was based on pilot studies of Gardner, Wansink (1) and Salmon, Fennis (2), showing that two products within food pairs indeed differed on palatability and healthiness. The 5 ‘rewarding’ high sugar/fat snack-type products that we selected were chocolate cookie, two types of crisps, chocolate bar, and waffle, and the other 5 more neutral products were unsalted peanuts, muesli bar, rice waffles, pear and apple (both groups of 5 products were matched on savoury and sweet foods). To further validate the classification of the selected products, a pilot study was conducted in a separate sample of 44 participants (29.5% male) with a mean age of 30.98 (*SD* = 8.75). Most participants were British (43.2%) and English (25.0%). The sample size was based on a power calculation in which we aimed to achieve a power of 0.90 and estimated a medium effect size of 0.5. Participants who were fluent in English were recruited via Prolific and received £0.45 upon completion of the study. Pictures of food products were obtained from the food image data base as developed and validated by Blechert, Lender (3) and we checked the selection of foods against the palatability ratings performed in this original study that used a sample of N = 1988 individuals ranging in age and weight from German speaking countries and North America After providing informed consent, they evaluated the palatability with two items (“How much do you like the taste of the product?” and “How much do you enjoy eating the food product?”) and the healthiness with two items (“How healthy to you think the food product is?” and “How nutritious do you think the food product is?”) of the 10 food products on 7-point scales ranging from 1 (not at all) to 7 (very much) (based on Gardner et al., 2014 and Salmon et al., 2014).
 See Table 1 for means and standard deviations of the ratings of each product. Because the apple was perceived almost as palatable as some of the rewarding foods, and the aim was to make a clear distinction between the groups of products, we decided to eliminate this product from the choice set. Consequently, to retain an equal amount of products in each group, also waffle was removed as this product was scored as the least palatable and most healthy option of the rewarding food products. Paired t-test showed that the group of 4 rewarding foods were on average indeed perceived as more palatable (*M* = 5.99, *SD* = 1.14) than the group of 4 neutral foods (*M* = 4.25, *SD* = 1.20), *t* (43) = 6.49, *p* < .001, *d* = 1.49, and as less healthy (*M* = 1.81, *SD* = 0.64) than the healthy foods (*M* = 4.63, *SD* = 0.88), *t* = *t* (43) = -20.32, *p* < .001, *d* = 3.67.

**S2. Table A**. Mean scores (SD) of each of the food products in the Pilot Study Food Rating

| Rewarding food group | Palatability | Healthiness | Healthy food group | Palatability | Healthiness |
| --- | --- | --- | --- | --- | --- |
| Chocolate cookie | 6.10 (1.35) | 1.92 (0.84) | Unsalted peanuts | 4.51 (1.99) | 4.19 (1.33) |
| Crisps 1 | 6.09 (1.45) | 1.83 (0.85) | Muesli bar | 4.39 (1.79) | 4.20 (1.45) |
| Crisps 2 | 5.89 (1.48) | 1.82 (0.82) | Rice waffles | 3.41 (2.00) | 6.13 (0.97) |
| Chocolate bar | 5.90 (1.58) | 1.67 (0.66) | Pear | 4.70 (2.08) | 6.13 (0.97) |
| Waffle | 5.53 (1.62) | 2.22 (1.06) | Apple | 5.47 (1.51) | 6.18 (0.95) |

Note: Scored on a 7-point scale. Apple and waffle were removed from the food groups in the online food shopping task.

**S2 References**

1. Gardner MP, Wansink B, Kim J, Park S-B. Better moods for better eating?: How mood influences food choice. J Consum Psychol. 2014;24(3):320-35.

2. Salmon SJ, Fennis BM, de Ridder DT, Adriaanse MA, De Vet E. Health on impulse: when low self-control promotes healthy food choices. Health Psychol. 2014;33(2):103-9.

3. Blechert J, Lender A, Polk S, Busch NA,Ohla K. Food-Pics_Extended—An image database for experimental research on eating and appetite: Additional images, normative ratings and an updated review. Front. Psychol. 2019;10(307).
