# Supplementary material for: The effect of personal relative deprivation on food choice: An experimental approach
Source: PLoS One. 2022 Jan 13;17(1):e0261317. doi: 10.1371/journal.pone.0261317 (PMC8758004; doi:10.1371/journal.pone.0261317)
Supplement: S3 Appendix — (DOCX) [file pone.0261317.s003.docx]

**S3 Appendix.**

**Results “bogus” task**

Participants in the PRD condition did not differ from the control condition in the amount of points that they allocated to healthy foods (radishes and carrots) or unhealthy foods (waffles and pralines), see S3 Table A.

**S3 Table A. Points allocated to healthy foods and unhealthy foods in the bogus task**

|  | Mean (SD) | |  |  |
| --- | --- | --- | --- | --- |
|  | PRD condition | Control condition | *t*(170) | *p* |
| Healthy foods | 7.67 (8.01) | 7.39 (7.63) | .23 | .82 |
| Unhealthy foods | 22.33 (8.01) | 22.61 (7.63) | .23 | .82 |
