# Supplementary material for: The effect of personal relative deprivation on food choice: An experimental approach
Source: PLoS One. 2022 Jan 13;17(1):e0261317. doi: 10.1371/journal.pone.0261317 (PMC8758004; doi:10.1371/journal.pone.0261317)
Supplement: S4 Appendix — (DOCX) [file pone.0261317.s004.docx]

**S4 Appendix.**

**Pilot study: Testing PRD manipulation and food choice task**

The primary aim of this non-preregistered pilot was to test the feasibility of the PRD manipulation and a food shopping task in a community sample. The secondary aim was to test whether PRD affected food choice.
 **Participants and procedure**Participants were recruited via posters and flyers during an open-campus day which was especially held for children and their parents. Inclusion criterion was a minimum of 18 years old. In total, 118 participants participated of which 17 were excluded (seven had an allergy related to the foods, and ten were accompanied by their child or partner). Hence, the analytic sample consisted of 101 participants (*M*_age_ = 43, SD = 12.48, 61% female) who were compensated with the three food products that they chose as part of the experiment. The experiment was advertised as a game to gather groceries. Experiments were run from 10.00 to 16.00 h on a walk-in basis in a computer room. After providing informed consent, they answered questions about their age, gender, hunger level, healthy eating and restraint goals. Next, they played the card game after which they filled in the Experienced PRD scale (see Study 1). The points earned with the card game were used as resources to ‘purchase’ three food products in an online food shopping task, where it was explicitly stated that they had to choose foods that they desired most at that moment and that they would receive them immediately after the task. The foods shown in the shopping task were pictures taken from the actual products. Based on the *Pilot study food rating* (see Study 2), the unhealthy rewarding options were chocolate cookie, two sorts of crisps, chocolate bar, and waffle, and the healthy, neutral options were apple, pear, rice waffles, muesli bar, and unsalted nuts. All items were presented as one serving (e.g. a little bag of crisps, one pear, a portion package of 5 thin rice waffles). After the food selection, participants were instructed to walk to an adjacent room where they received the food products and the debriefing. The ethical committee of social sciences of the university approved the study.

**Results**

**Correlations and comparability**There was a significant correlation between gender and unhealthy food choice, Spearman’s rho = .25, *p* = .013. A chi-square analysis indicated that women were more likely to choose unhealthy foods than men, x ^2^ =3.27, *p* = .07. Age was negatively correlated with unhealthy choices, Spearman’s rho = - .31, *p* = .002.
 Conditions did not differ in pre-test variables hunger, age and gender. They did differ on one of the healthy eating / dietary restraint items, the PRD condition (*M* = 5.87, SD = .82) had a higher healthy eating goal than the control condition (*M* = 5.47, SD = .96), t(99) = -2.24, *p*  = .028. Hence, age, gender, and healthy eating goal were used as covariates in the test of hypothesis.

**Manipulation check**The PRD condition (*M* = 5.04, SD = .96) experienced more PRD than the control condition (*M* = 1.99, SD = 1.09) (on a 7-point scale), *t*(99) = -14.91, *p*  <.001, so the manipulation appeared succesful.

**Test of hypotheses**An ANCOVA of PRD on unhealthy food choices, controlling for age, gender and healthy eating goal, revealed no main effect, *F*(1,96)= 0.08, *p* = .78. There was no difference in choosing unhealthy foods between the control condition (*M _adj_* = 1.21, SE = .13) and PRD condition *(M _adj_* = 1.16, SE = .14)

**Exploratory analyses**For exploratory purpose, interactions were tested between condition and the control variables using bootstrapping in PROCESS (1). Controlling for age, gender, and healthy eating goal, a significant interaction between condition and hunger was found, *F*(1, 94) = 4.61, *p* = .03, *R^2^*-change = 0.04. Simple effects of condition on food choice were non-significant at low level of hunger (-1SD), *B* = 4.46, *t*(94) = 1.59, *p* = .11, CI[-0.11, 1.03], moderate level of hunger (+1SD), *B* = -.47, *t*(94) = -1.38, *p* = .17, CI[-0.90, 0.16]. Testing this interaction without two participants who scored the highest on hunger, i.e. 6 on a 7-point scale, revealed a non-significant interaction effect between hunger and condition on rewarding food choice controlling for the covariates, *F*(1, 92) = 2.93, *p* = .09, *R^2^*-change = 0.04. Other interaction effects between condition and control variables were all non-significant, *p* > .08.

**Discussion**

The manipulation appeared again succesful, but no main effect of condition on rewarding food choices was found. Exploratory analyses revealed that condition interacted with hunger level. Although not significant, a trend was observed that the PRD condition chose more rewarding foods when low in hunger level. This pilot study had considerable methodological limitations. Participants selected foods from a computer screen and the idea of receiving the selected foods from the researcher may have influenced their selection. Also, as participants entered (and left) the computer room at any time during the open-campus event, some bringing their children, it was a non-ideal, rather chaotic setting for a laboratory experiment.

**S4 References**

1. Hayes AF. Introduction to mediation, moderation, and conditional process analysis: A regression-based approach: Guilford publications; 2017.
